# A new therapy to alleviate the inflammatory injury of piglet intestine caused by short-distance transportation——music

**DOI:** 10.1371/journal.pone.0313354

**Published:** 2025-02-25

**Authors:** Qin Fu, Bin Bai, Sitong Zhou, Yunlong Zhao, Yue Yang, Xiaohong Zhang, Xuanning Liu, Mengyao Wu, Wenzhong Zhao, Jun Bao, Honggui Liu

**Affiliations:** 1 College of Animal Science and Technology, Northeast Agricultural University, Harbin, Heilongjiang, P.R. China; 2 Institute of New Rural Development, Harbin, Heilongjiang, P.R. China; 3 Key Laboratory of Swine Facilities Engineering, Ministry of Agriculture and Rural Affairs, Harbin, Heilongjiang, P.R. China; University of Wisconsin-La Crosse, UNITED STATES OF AMERICA

## Abstract

The purpose of this study was to explore whether music can reduce stress in animals by regulating the activity of the hypothalamus pituitary adrenal cortex (HPA) axis and reducing the concentration of cortisol. The control group was not played with any music or mechanical noise, the music group played music before and during transport, and the noise group played noise before and during transport as a positive control. The results showed that after two-hours of transportation, the concentrations of adrenocorticotropic hormone and cortisol in the music group were lower than that in the control and the noise groups, while the cortisol level in the noise group was higher than that in the music and the control groups. Plasma concentrations of D-lactic acid and diamine oxidase in the music group were lower than those in the control group and the noise group, and the noise group was higher than the control group. In addition, the concentrations of jejunal inflammatory factors interleukin-6 and interleukin-8 in the music group were lower than those in the control group and the noise group, but there was no difference of interleukin-12 in the three groups. However, there was no significant difference in Illinois-6, Illinois-12 and INF-γ between the noise group and the control group. The contents of reactive oxygen species, malondialdehyde and glutathione peroxidase in the music group had no changes compared with those in the control group, while the contents of reactive oxygen species and malondialdehyde in the noise group were higher than the control and the music groups, and the content of glutathione peroxidase was decreased. Compared with the music group, differently expressed genes analysis also showed that the mRNA expression level of inflammatory genes in the jejunum of the music group’ piglets decreased. In addition, compared with the music group, some Kyoto Encyclopedia of Genes and Genomes (KEGG) pathways related to inflammation were highly expressed in the control group. In conclusion, our results showed that musical stimulation can reduce the inflammatory response of piglets caused by transportation by reducing the activity of HPA axis. Noise increased the activity of HPA axis, which aggravated the intestinal damage of piglets and caused intestinal oxidative damage

## Introduction

The transportation of weaned piglets is a common practice. Pigs are mainly transported to separate production facilities when weaning, so as to reduce the vertical spread of diseases and improve the early growth and production potential of pigs after weaning [1]. During transportation, pigs are exposed to various stressors, which causes stress reactions in pigs. The intestinal tract is the earliest and most vulnerable organ of the body under stress, and it has been widely recognized that stress causes intestinal injury. Under stressful conditions, it will lead to congestion in the intestinal mucosa, intestinal villi damage, intestinal barrier damage, and promote intestinal inflammatory reaction [2]. The intestinal tract is the largest organ of digestion, absorption and immunity in the body, and the jejunum plays a major role in the digestion and immunity of small intestine. In our previous research, it was found that short-distance transportation for 2 hours increases the overall stress level in piglets by jejunal ROS content and inflammation, increases the content of ROS in the jejunum and cause the jejunum inflammation [3].

It is particularly important to find a way to reduce the transportation stress of piglets and improve the transportation welfare of piglets in livestock production. It has been proved in humans that listening to music can reduce anxiety, stress and patients’ tolerance to surgery, and has a beneficial effect on inflammatory markers [4]. Bloomsmith et al. (1991) [5] believe that sensory stimulation can serve as an environmental enrichment method. With the continuous deepening of music research, music as a way to enrich the environment is increasingly valued by zootechnics and veterinary medicine. Music environment stimulation is often used as an effective and easy to implement treatment measure to alleviate animal anxiety levels and calm their tense emotions [6]. Music is a non-pharmacological, low-risk treatment measure that has low workload for farms and it is a research-worthy treatment. Music, as a way of enriching the environment, has been reported to improve the welfare and health of animals. For example, in a new sinking experiment, zebrafish (*Danio rerio*) listening to music showed less anxiety and lower levels of peripheral pro-inflammatory cytokines [7]. Male rats exposed to Mozart k.488 music showed decreased anxiety and depression-like behaviors, and increased density of hippocampal spine density [8]. Music can also reduce the stress response of dogs, cats and monkeys, and make them calmer [9]. Exposure to a noisy environment will stimulate the stress response of the body, the accumulation of ROS, oxidative damage, adversely affect immune parameters, and reduce immunity [10]. At present, it is generally believed that music can reduce the stress level of pigs. Studies have shown that music can improve growth performance and relieve weaning stress of piglets [11]. There are few studies on the effects of music on transportation stress. Li et al. (2021a) [10] showed that short-term music stimulation can help to alleviate stress. After a 5-day music adaptation period, playing the same music during the transportation of piglets has a positive effect on reducing the stress and fighting behavior during the transportation [12].

Noise is a pervasive, harmful and unpleasant auditory stimulus and it is a common stress source with adverse effects on human and animal health [13]. The external ear shape of pigs is particularly large and the ear cavity is deep. In fact, pigs have very developed hearing, and they can keenly detect very weak sounds. Noise is a particularly important factor in reducing animal welfare. Noise can cause physical and psychological discomfort in mammals [14,15]. Noise in the process of animal husbandry production may accelerate the respiratory frequency and heart rate of pigs. This will lead changes in performance and physiological response, such as reducing the fertility level and meat quality level of pigs, and leading to the disorder of endocrine system and the decline of immunity. In the study of Kanitz et al. (2005) [16], 12-week-old piglets were exposed to noise for 4 weeks. The results showed that noise would cause a series of changes in adrenocortical hormone and cortisol hormone, which first increased, then decreased and then increased [16]. This experiment further proved that the pig house at the noise environment might bring more pressure to pigs. In addition, whether it is intermittent or continuous noise, it may bring adverse consequences to the welfare of pigs.

The hypothalamic pituitary adrenal (HPA) axis is an important neuroendocrine axis in the body, which can participate in and control stress responses. The HPA axis is characterized by regulating the hormone cascade response of glucocorticoids. Under stress, HPA axis is activated, causing CRH stimulates the release of adrenocorticotropic hormone by the anterior pituitary gland and ACTH can further stimulate the adrenal cortex to release cortisol. Study has shown that a stress induced increase in cortisol can cause intestinal inflammation through the brain-gut axis, and stress can lead to rapid degradation of intestinal structure, induce intestinal villi atrophy, and increase the chance of secondary infection through impaired intestinal barrier function [17]. In previous studies, researchers have shown that music can reduce the concentration of cortisol by regulating the activity of the HPA axis, thereby reducing the body’s stress response [18]. In addition, music can also alleviate negative emotions in animals. Musical environment stimulation is often used as an effective and easily implemented therapeutic measure to reduce anxiety level and tension state of animals. Music can reduce cortisol by regulating HPA axis activity. Cortisol mainly acts on the prefrontal lobe and marginal structures, which is closely related to cognitive and emotional regulation [19]. This study played the same music before and during transportation of piglets, assuming that music can reduce the concentration of cortisol by regulating HPA axis activity, thereby alleviating intestinal inflammatory damage caused by transportation stress.

## Materials and methods

All experiments and procedures carried out in this study were approved by the Animal Protection and Utilization Committee of Northeast Agricultural University (NEAUEC20200346), and were conducted under the guidelines of the “Rules of Animal Protection and Utilization of Northeast Agricultural University”.

### Piglets and Housing

A total of 108 weaned piglets (Large White×Landrace), 6 weeks old and with similar body weight, were selected as experimental animals and randomly divided into three treatment groups: the control group (CON group), the music group (MUS group) and the noise group (NOI group). The three groups of piglets were raised in three large enclosures, each consisting of six repetitive small pens with the same structure. In short, there were six replicate pens per group, six pigs per pen (1.58 ×  1.71 m). Each group had 36 piglets, and there were 108 pigs in three groups altogether. Every pen was insulated with soundproof cotton. The temperature was controlled at 20-25°C and the humidity was controlled at 65% -75%. Piglets were raised for a week, during which they freely consumed conservation feed and water. The conservation feed was based on corn/soybean, and the composition of this feed is shown in S1 Appendix. During the feeding period, the environmental noise was measured by a high-precision noise meter (Xima 30-130dB) to be about 40dB. The CON group was not played with any music or mechanical noise, the MUS group was played classical music (Mozart K.488) with a music loudness modulation of 65dB, and the NOI group was played previously recorded original house background noise (mechanical noise, such as fans) with a noise intensity setting of 85 decibels. Music and noise played from 10:00 am to 16:00 PM, a total of 6 hours of rotation.

### Transportation phase

All piglets were fasted but allowed to drink water at 21:00 the night before transportation. Three constant temperature transport trucks were used to transport piglets. Before transportation, the trucks were disinfected and Bluetooth music players were installed on the vehicles in advance. The truck has two layers, each with 6 fences (long×Width:210 × 110 cm), with an area of about 2.31 m^2^. The three groups of piglets were loaded to the bottom of the three trucks with 12 piglets per fence, and the transport density was about 0.19 m^2^/piglet. The metal floor of the fence was covered with 2 cm thick straw, and water and feed were not provided throughout the transportation process. During transportation, piglets were exposed to music (the MUS group) or noise (the NOI group) using remote control consistent with that during feeding, while the CON group piglets were not exposed to music or noise. The transportation started at 9am, with a temperature of around 25 °C and a humidity of 60% inside the truck. The piglets were transported in the truck by three experienced drivers, along a flat road, for a relatively short distance (120 km) for 2 hours (average speed, 60 miles/hour). After the transportation, one piglet was randomly selected from each pen with 6 pens in each group, for a total of 18 piglets euthanized from the three groups were euthanized. Blood was collected from each of the pigs’ hearts, centrifuged them to obtain serum and plasma, and then put them in a refrigerator at -80°C for analysis. At the same time, the jejunum was quickly separated in the ice bath, and a part of it was cut into 1 cm^3^ small pieces and put into a tube containing 4% paraformaldehyde for the analysis of intestinal morphology. In addition, a part of the jejunum was cut and put into RNase-free eppendorf tube for the detection of the jejunum physiological indexes.

### Detection of the jejunum morphology of piglets after transportation

The jejunum tissue samples fixed with 4% paraformaldehyde were dehydrated with ethanol, gradually replaced with xylene, and then embedded in paraffin. The embedded tissue was cut into 6μm thick slices, pasted on the glass slide and dried. Then, the slices were dewaxed, washed with tap water, dehydrated with ethanol, stained with Eosin for 5 minutes, dehydrated again, and sealed [3] (Fu et al., 2023a). Then, images were collected and analyzed by a microscope. Scanning software (CaseViewer2.4) was used to select the tissue target area and perform 50x imaging magnification. When imaging, it was necessary to make the tissue cover the whole field of vision as much as possible, and to ensure that the background light of each photo was consistent. After the imaging was completed, Image-Pro Plus 6.0 analysis software was used to measure the length of 5 complete intestinal villi and the corresponding depth of 5 crypts in each section with millimeter as the standard unit, and the ratio of intestinal villi to crypts was calculated.

### Detection of HPA axis hormone indexes

The serum was taken out of the refrigerator and thawed in water, and then the concentrations of ACTH, cortisol and norepinephrine were detected according to the operation steps of ELISA kit (Nanjing Jiancheng Bioengineering Institute, Nanjing, China; Intra-batch difference: CV < 10%; Batch difference:CV < 5%). Firstly, standard samples were prepared for each indicator to quantify the content of the target indicator in the tested sample. Then, used an enzyme-linked immunosorbent assay (ELISA) to measure the absorbance of the standard substance and the tested sample at the wavelength specified in the instruction manual. The absorbance of the standards were used to draw a standard curve, and calculated the concentration of each indicator in the piglet serum sample through the standard curve [3].

### Detection of plasma DAO and DLA

According to the rate method, the content of DAO was determined by biochemical kit. Eighty microliters of plasma were added into the test tube, and then took out 800ul of the reagent from the biochemical kit. Immediately added it into the test tube and mixed it evenly. The mixed reagent was quickly added into the colorimetric quartz dish, placed on the ultraviolet spectrophotometer (340nm), waited for 20s, read the absorbance, and recorded as A1 value. The liquid was then poured into the previous test tube and placed in a water bath at 37°C for 10 minutes. Then it was quickly poured into the quartz dish, and the absorption value was read again at 10 minutes and 20 seconds, and was recorded as the A2 value. Then the activity value of DAO was calculated by the formula. DAO (U/L) =  (A1-A2)/ (6.3 x colorimetric light diameter (0.5 cm) x reaction time (10 minutes)) x total volume of reaction liquid (880ul)/ Sample size (80ul). The measurement method of DLA was similar to that of DAO. Placing the spectrophotometer at 450nm, added the corresponding reagent in the colorimetric dish according to the kit requirements, and then recorded a blank value as A1. Measuring another measured value as A, the difference between A and A1 was marked as △A. The DLA content was calculated according to standard curve Standard curve: y =  39.127x - 0.0101; (x: the standard molar mass (μmol), y: △A), DLA content (µmol/mL) = 0.43 × (△A + 0.0101) × 0.06mL. The detection methods of DAO and DLA referred to the detection methods of Wang et al. (2020) [20]. The kits: Nanjing Jiancheng Research Institute (intra batch difference: CV < 8%; inter batch difference: CV < 10%).

### Determination of inflammatory factors and antioxidant enzymes in the jejunum

0.5g of jejunal tissue were weighed, and rinsed it in pre cooled PBS buffer to remove residual blood. The tissue was then wiped dry with filter paper and weighed.The swabbed tissue was then dried and weighed and placed in a 10 ml beaker. The pre-cooled PBS buffer of 9 times the weight of the tissue was weighed and added to the beaker. The tissue was cut and homogenized with a hand-held homogenizer. The prepared homogenate was centrifuged for 10 minutes at 2000r/min, and the supernatant was collected. The contents of malondialdehyde (MDA), glutathione peroxidase (GSH-PX), interleukin 6 (IL-6), interleukin 8 (IL-8) and interleukin 12 (IL-12) were determined according to the operation steps of Nanjing Jiancheng ELISA Kit (intra batch difference: CV < 8%;inter batch difference:CV < 10%).

### Reactive oxygen species (ROS) detection

Firstly, intestinal slices were thawed and dried. Then, the grouping pen was used to draw circles around the tissue. Reactive oxygen species (ROS) dye solution was added drop by drop and incubated in the incubator at 37°C for 30min away from light. The slides were washed three times for 5min each time. Drying slightly, the DAPI dye solution was added in the circle, and incubated at room temperature for 10min away from light. The slides were washed three times for 5min each time again. The slides were dried and sealed with anti fluorescence quenching sealing agent. Finally, the slides were observed under the fluorescence microscope and the images were collected. DAPI has an ultraviolet excitation wavelength of 330-380nm and an emission wavelength of 420nm. DAPI stained nuclei were blue under UV excitation, and the positive expression was red light labeled with corresponding fluorescent. The image pro plus 6.0 software was used for analysis, and the accumulation of three frontal visual fields, optical density (IOD), corresponding pixel area (area) and tissue area density (area density) of each slice were calculated [21].

### The jejunum transcriptome sequencing

Total RNA was extracted from the jejunum of piglets with Trizol (Invitrogen, Carlsbad, CA, USA) reagent, and purified to obtain clean messenger RNA (mRNA). Then the mRNA was decomposed into small fragments (200-300 bp in length). Subsequently, the first strand complementary DNA (cDNA) and the second strand cDNA were synthesized. The whole transcriptome analysis step was completed by China Hangzhou Lianchuan Biotechnology Co., Ltd. In this study, Illumina hiseqtm high-speed sequencer was used to screen and sort out the sequencing data. DESeq2 was used to analyze the differentially expressed genes with |  log2 (Fold Change) |  greater than 1.5 and P value less than 0.05. Subsequently, the Kyoto Encyclopedia of Genes and Genomes (KEGG) enrichment analysis was performed on all differentially expressed genes (DEGs).

### Real-time quantitative polymerase chain reaction

Primer premier 5.0 software was used to synthesize and design specific primer related gene primer sequences for PIK3 CD, LYN, VAV1, CD22, IRAK2 and FOXO1 genes. These six genes were used for the validation of DEGs in KEGG pathway. Trizol reagent (Invitrogen, Carlsbad, CA, USA) was used to extract total RNA from the jejunum. Cfx384 touchtm (Bio Rad, USA) was used for real-time fluorescent quantitative PCR (QRT PCR). The reaction system of QRT PCR was 10 μL, including 5 μL fluorescent dye, 1 μL template dilution, 0.3 μL upstream and downstream primers (10 μM) and 3.4μL double distilled water. The expression level of beta-actin was used as an internal reference for standardization, and the relative expression level of each gene mRNA was calculated using the 2^-△△^^CT^ method. The primer sequences of the target genes are shown in Table 1.

**Table 1:** Primer sequence of target gene.

GENE	FORWARD PRIMER (Five,-Three,)	REVERSE PRIMER (Five,-Three,)
PIK3 CD	TCAACAAGGATGCTCTGCTCAACTG	CAGGATGGCATAGGCTCGTTCAC
LYN	ACCAAGGAGGAGCCCATCTACATC	GCGATCTGAGCGGAGAAGTCAATC
VAV1	GCTCAAATACCACCTCCTTCTCCAG	GCCGCAAGTTCTCCTTCTCCATC
CD22	ACGGCAGTCCTGTCCTGTGAG	CTGATGGTAGTGGTGGATGTCTTGG
IRAK2	CTGCCACCTCAACATCTTACCTCTG	TGAGCCACCCTGACCCTGAAG
FOXO1	TGTCCTACGCCGACCTCATCAC	GCACGCTCTTGACCATCCACTC

### Data statistics and analysis

The data was analyzed by IBM SPSS statistical software (version 22.0, SPSS Inc., USA). In our study, all data were tested for compliance with a normal distribution, with the experimental unit being a fence. All results showed a normal distribution. Then we used independent sample t-tests to test significance of the date of gene verification. Meanwhile one-way analysis of variance was used to analyze the differences among the three treatment groups, including all the remaining detection indicators. All results were represented as mean and standard error (Mean and SEM). A *P*-value < 0.05 was considered significant. * Represents significant differences between different groups ( * *P* < 0.05, *  *  *P* < 0.01).

## Results

### The effect of music treatment on the jejunal barrier in piglets

Intestinal villus height, crypt depth and ratio of villus to crypt are important indicators to evaluate intestinal morphology, which can reflect intestinal function and health status to a certain extent. Table 2 shows the effect of short distance transportation on the morphology of the jejunum of piglets. After two hours’ transportation, the villus height, crypt depth and ratio of villus to crypt of the jejunum of control CON group piglets were not significantly different from those of MUS group piglets (*P* > 0.05), while the height and ratio of villus to crypt of the jejunum of NOI group piglets were significantly lower than those of CON group piglets and MUS group piglets (*P* < 0.001), There was no significant difference in the depth of jejunal crypt among the three groups.

**Table 2:** Effects of music and noise treatment on the morphology of the jejunum of piglets after short-distance transportation.

INDEX	TREATMENT GROUP			P-value
	CON	MUS	NOI	
Villus length(mm)	0.385^a^ ± 0.003	0.389^a^ ± 0.002	0.343^b^ ± 0.002	< 0.001
Crypt depth(mm)	0.147 ± 0.003	0.146 ± 0.004	0.147 ± 0.005	0.766
Ratio of cashmere to cryptochrome	2.619^a^ ± 0.041	2.664^a^ ± 0.003	2.333^b^ ± 0.011	< 0.001

Different superscripts of

^a^and

^b^is a method of expressing differences, and it is necessary to compare whether the letters represented by the two groups overlap. Different superscripts of ^a^ and ^b^ indicate significant differences between different groups, *P* <  0.01. The same letter or no letter indicates that there is no significant difference.

### The effect of music treatment on plasma DLA and DAO concentrations in piglets

Table 3 shows the effects of transportation on the concentrations of DLA and DAO in plasma of piglets stimulated by music and noise. The plasma concentrations of DLA and DAO in MUS group were significantly lower than those in CON group and NOI group (*P* < 0.001). The concentrations of DLA and DAO in plasma of piglets in NOI group were significantly higher than those in CON group and MUS group (*P* < 0.001).

**Table 3:** Effects of music and noise treatment on plasma DLA and DAO of piglets after short-distance transportation.

INDEX	TREATMENT GROUP			P-value
	CON	MUS	NOI	
DLA (µmol/mL)	0.560^b^ ± 0.321	0.447^c^ ± 0.270	0.810^a^ ± 0.545	< 0.001
DAO (U/L)	53.777^b^ ± 2.224	48.603^c^ ± 2.928	72.565^a^ ± 5.063	< 0.001

Different superscripts of

^a^and

^b^is a method of expressing differences, and it is necessary to compare whether the letters represented by the two groups overlap. Different superscripts of ^a, b^ and ^c^ indicate significant differences between different groups, *P* <  0.01. The same letter or no letter indicates that there is no significant difference.

### Effects of music treatment on cortisol and cytokines of piglets after short distance transportation

Table 4 shows the effect of short distance transportation on serum HPA axis hormones of piglets in CON group, MUS group and NOI group. The serum ACTH and cortisol concentrations in the MUS group were significantly lower than those in the CON and NOI groups (*P* <  0.001). The NOI group was significantly higher than CON group and MUS group (*P* <  0.001). At the same time, table 4 shows the effect of transportation on the piglet jejunal inflammatory factors in three groups. Concentrations of jejunal inflammatory factors IL-6 and IL-8 were significantly down-regulated in the MUS group compared with the CON and NOI groups (*P* <  0.001), there was no significant difference in IL-12 among the groups (*P >* 0.001). There was no significant difference in IL-6, IL-8 and IL-12 between NOI group and CON group (*P* >  0.05).

**Table 4:** Effects of music and noise treatment on physiological indexes of piglets after short-distance transportation.

INDEX	TREATMENT GROUP			P-value
	CON	MUS	NOI	
CORTISOL (mg/L)	110.167^b^ ± 4.540	92.1294^c^ ± 2.190	124.403^a^ ± 3.347	< 0.001
ACTH (ng/L)	73.068^b^ ± 2.072	61.109^c^ ± 3.087	86.539^a^ ± 2.512	< 0.001
IL-6 (pg/ml)	440.763^a^ ± 6.546	329.780^b^ ± 8.116	435.916^a^ ± 5.900	< 0.001
IL-8 (pg/ml)	43.777^a^ ± 2.811	28.120^b^ ± 2.213	44.677^a^ ± 1.703	< 0.001
IL-12 (pg/ml)	322.812 ± 12.252	325.331 ± 7.716	322.607 ± 8.054	0.679

Different superscripts of

^a^and

^b^is a method of expressing differences, and it is necessary to compare whether the letters represented by the two groups overlap. Different superscripts of ^a, b^ and ^c^ indicate significant differences between different groups, *P* <  0.01. The same letter or no letter indicates that there is no significant difference.

### Effects of music and noise treatment on jejunal oxidase in piglets after transportation

**Fig 1:**
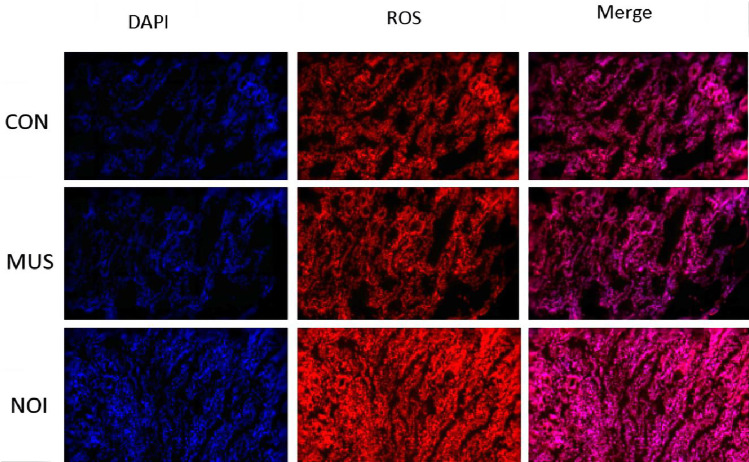
Effects of music and noise treatment on ROS in the jejunum of piglets after transportation. **(A)** High-quality fluorescent ROS.

Table 5 shows that the contents of ROS, MDA and GSH-Px of reactive oxygen species in MUS group have no significant changes compared with CON group (*P* > 0.05). The content of ROS and MDA in the NOI group was significantly higher than that in the CON and MUS groups (*P* <  0.001), but the content of GSH-Px (*P* <  0.001) decreased significantly. Figure 1 shows the fluorescent picture of the effect of music and noise treatment on ROS in the jejunum of piglets after transportation. Among them, DAPI dye solution is blue when it binds to the nucleus, and ROS positive is red. The figure of Merge is the synthetic picture of the first two. As shown in table 1, the content of ROS in the NOI group was significantly higher than that in the CON and MUS group.

**Table 5:** Effects of music and noise treatment on jejunal oxidase in piglets after transportation.

INDEX	TREATMENT GROUP			P-value
	CON	MUS	NOI	
MDA (nmol/g)	46.758^b^ ± 1.374	47.163^b^ ± 1.163	60.036^a^ ± 2.623	< 0.001
GSH-PX (U/g)	476.380^a^ ± 9.670	474.605^a^ ± 12.235	426.629^b^ ± 4.650	< 0.001
ROS (Positive cell density)	18.179^b^ ± 0.624	17.831^b^ ± 0.419	26.742^a^ ± 0.617	<0.001

Different superscripts of

^a^and

^b^is a method of expressing differences, and it is necessary to compare whether the letters represented by the two groups overlap. Different superscripts of ^a^ and ^b^ indicate significant differences between different groups, *P* <  0.01. The same letter or no letter indicates that there is no significant difference.

### DEGs analysis

**Fig 2:**
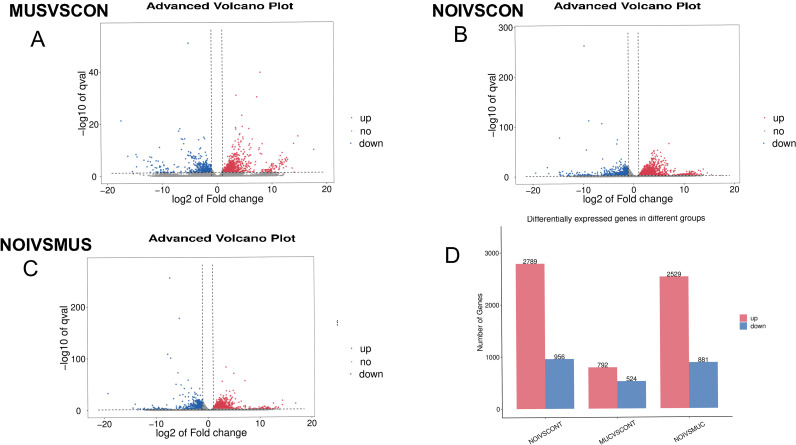
Effects of Music and Noise Treatment on DEGs in the jejunum of piglets after short-distance tansportation (A, B, C). Volcanic map of all differentially expressed genes. Red represents significantly up-regulated genes, blue represents significantly down regulated genes, and gray dots represent genes with insignificant differential expression. (D) Histogram of the number of differentially expressed genes between the transportation stress group and the control group. Red genes are up-regulated and blue genes are down-regulated.

In order to investigate changes in gene levels in the jejunum as a result of music treatment, DEGs in the jejunum were determined by transcriptome sequencing. Fig 2 shows the effect of music and noise treatment on piglet jejunal DEGs after short distance transportation. In this study, a total of nine samples were transcribed and sequenced from the CON group, the MUS group and the NOI group. The overall quality of RNA-seq data was good, which met the basic sequencing requirements. According to the volcanic map, there were 1316 differentially expressed genes (Fold change (FC) ≥ 2 or fold change ≤ 0.5 and p < 0.05) in MUS group compared with CON group, of which 792 genes were up-regulated and 524 genes were down-regulated (Fig.2A). Compared with CON group, there were 3745 differentially expressed genes in NOI group, 2789 genes were up-regulated and 956 genes were down regulated in volcanic map (Fig.2B). Compared with the MUS group, there were 3410 differentially expressed genes in the NOI group, of which 2529 genes were up-regulated and 881 genes were down regulated in the volcanic map (Fig. 2C). Figure 2D showed the histogram of the differential genes.

### KEGG enrichent analysis of DEGs

**Fig 3:**
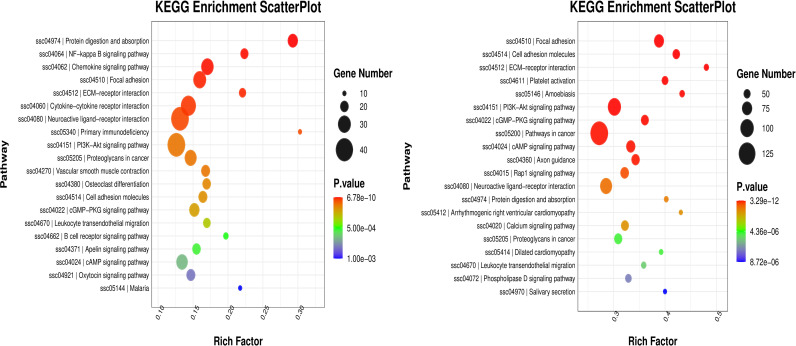
Effects of Music and Noise Treatment on KEGG pathway in the jejunum of piglets after short-distance transportation. The first 20 KEGG pathways are analyzed. The x-axis represents the degree of enrichment. In the scatter diagram, the size of points represents the number of genes, and the color of points represents the p value of enrichment analysis. (A) showed the KEGG_ bubble chart of the MUS group vs the CON group. (B) showed the KEGG_ bubble chart of the NOI group vs the CON group.

In order to better understand the gene functions and gene products involved in music and noise, KEGG database was used to annotate the functions of differentially expressed genes in jejunal tissue cells to determine their biological functions. KEGG enrichment bubble chart (Fig 3) is the path of top 20 with the smallest p value (or Q value) for display. The inflammation-related KEGG pathways ‘NF−kappa B signaling pathway’, ‘PI3K-Akt signaling pathway’ and ‘B cell receptor signaling pathway’ in the jejunum of piglets in the MUS group and the CON group were enriched. KEGG pathways such as ‘PI3K−Akt signaling pathway’ and ‘cancer pathway’ in the jejunum of piglets in NOI group and CON group were enriched. According to these pathways, the typical genes of each pathway were selected for validation, and the validation results were displayed in qRT-PCR.

### Validation of real-time fluorescent quantitative PCR results

The mRNA expression levels of genes PIK3 CD, LYN, VAV1, CD22, IRAK2, FOXO1 and for transcriptome validation are shown in Table 6. As expected, the expression of these genes was significantly down regulated in the jejunum of MUS group. This result is consistent with the results of transcriptome analysis, which shows that our transcriptome analysis has better reliability.

**Table 6:** Validation of transcriptomics sequencing using qRT-PCR.

GENE	CON vs MUS				NOI vs CON			
	DIFFERENCE MULTIPLE		P-value		DIFFERENCE MULTIPLE		P-value	
	Transcription group^1^	qRT-PCR^2^	Transcription group^1^	qRT-PCR^2^	Transcription group^1^	qRT-PCR^2^	Transcription group^1^	qRT-PCR^2^
PIK3 CD	1.51	2.01	0.020^*^	0.003^**^	2.3	1.8	0.098^*^	0.032^*^
LYN	1.53	2.58	0.030^*^	0.005^**^	1.29	0.98	0.800^ns^	0.985^ns^
VAV1	4.31	3.58	< 0.001^**^	< 0.001^**^	1.18	1.10	0.688^ns^	0.891^ns^
CD22	3.21	5.12	< 0.001^**^	0.004^**^	1.57	2.51	< 0.001^**^	< 0.001^**^
IRAK2	1.45	2.31	0.043^*^	0.002^**^	1.50	1.42	0.082^*^	0.023^*^
FOXO1	2.91	3.47	0.006^**^	< 0.001^**^	1.01	1.13	0.249^ns^	0.586^ns^

^1^the value represents the average value of piglets in 6 pens. There are 6 piglets in each repeated pen (n = 6) and 6 pens in each group (n = 36), the transcriptome has three biological replicates, and each replicate is a mixture of two fenced piglet the jejunum samples.

^2^the value represents the average value of piglets in 6 pens. There are 6 piglets in each repeated pen (n = 6) and 6 pens in each group (n = 36), the biological duplication of transcriptome is 6.

SEM stands for standard mean error.

^ns^stands for no significant difference between the two groups before, *P* >  0.05.

## Discussion

### Effects of music and noise on HPA axis hormones in piglets

The hypothalamic-pituitary-adrenal (HPA) axis is a crucial neuroendocrine axis in the body, which can participate in the control of the stress response. The HPA axis is characterized by the regulation of glucocorticoid hormone cascade. Evaluating the activity of the HPA axis has become a standard method for assessing animal stress levels [22]. Corticotropin releasing hormone (CRH) system is the most fully studied system for regulating the stress response of the central and gastrointestinal tract [23]. The CRH system has been proved to mediate various stress-induced gastrointestinal changes, including intestinal permeability and inflammation [24,25].

It is worth studying how music processing can alleviate the intestinal injury caused by transportation. In previous studies, it has been proved that music can reduce the stress response of the body by regulating the activity of HPA axis and reducing the concentration of cortisol [18]. Cortisol is the main component of major glucocorticoid found in most mammals. L.Kremer et al. (2020) [26] believe that cortisol increases under stress, for example, under stress or pain, a higher value of cortisol is detected in the saliva of pigs. Consistent with this view, Rey-Salgueiro et al. (2018) [27] and others pointed out that transportation led to the increase of cortisol level in pigs before slaughter. They believed that cortisol proved a reliable indicator of high stress. Studies have shown that stress has an effect on the gastrointestinal immune system. Stress factors such as physical and psychological stress have been proved to increase gastrointestinal inflammation [28,29]. A research showed that music could prevent depression and anxiety- like behavior caused by stress in mice, and reduced cortisol levels in the hippocampus tissue [6]. In addition, music can relieve the anxiety of the arrested gorillas, thus relieving animal stress [30]. These studies have shown that music has alleviating effect on animals in stress or pathological state. This experiment also showed the same results. The concentrations of HPA axis hormones ACTH and cortisol in MUS group were significantly lower than those in CON group and NOI group, while the levels of ACTH and cortisol in NOI group were significantly higher than those in MUS group and CON group. The results of this study are consistent with previous studies, that is, short-term music stimulation can reduce the level of cortisol and reduce stress response. The short-term noise environment will increase the cortisol level, which is also confirmed by the research of Li et al [10]. Previous reports also indicated that animals exposed to noisy environments activated the HPA axis and increased cortisol levels. The results of this study suggest that Mozart’s music reduces stress response of transportation on the jejunum of piglets by decreasing HPA axis activity and reducing cortisol release. However, noise can increase the release of cortisol through the activation of HPA axis, which aggravates the stress response of transportation to the jejunum in piglets.

### Effects of music and noise treatment on jejunal inflammation and oxidative stress in piglets after transportation

During transportation, the intestinal structure changes, the intestinal mucosa is damaged, the level of reactive oxygen species rises sharply, and the intestinal immune system is attacked, which may further lead to intestinal inflammation and oxidative stress [2]. Music enriched environment can effectively improve the immune function of the body, and noise will have adverse effects on the immune parameters of the body [31]. Interleukin 6 (IL-6) stimulates the acute-phase inflammatory protein C-reactive protein (CRP) and is involved in many mechanisms, including apoptosis, and low-grade inflammation. Studies have shown that the increase of IL-8 can make neutrophils gather to the inflammatory site and infiltrate into the intestinal stromal tissue, leading to the injury of intestinal mucosa [32]. In this study, the concentrations of the jejunal inflammatory factors IL-6 and IL-8 were significantly lower in the MUS group than in the CON and NOI groups, whereas there was no significant difference in IL-12 between the groups. This study shows that music can improve the immune function of the body and reduce the inflammatory reaction caused by stress. This is consistent with previous studies that listening to music usually reduces interleukin-6 (IL-6) levels [33]. Related studies have found that Mozart music reduced the levels of interleukin 4,10 and 13 (TH2 cytokines) in allergic patients, and increased the levels of interferon γ (INF-γ) and interleukin-12 (TH1 cytokine) [34]. The results also showed that there was no significant difference of IL-6, IL-12 and interferon-γ between the NOI group and the CON group, indicating that short-term noise stimulation did not aggravate the inflammatory response of piglets. The results of this study are consistent with those of Li (2021a) [10] and others. Short term noise has no significant effect on inflammatory factors in pigs [10], previous studies have pointed out that long-term noise stimulation can induce inflammatory response and reduce the body’s immunity [35]. In conclusion, the results of this study demonstrate that Mozart music reduces the inflammatory response to transportation on the jejunum of piglets and that noise does not exacerbate the inflammatory response to transportation on the jejunum of piglets. Because music can reduce the concentration of cortisol and reduce the stress response of piglets, this study speculates that Mozart music can reduce the inflammatory damage of stress to the jejunum of piglets by reducing the stress of piglets.

Due to stress, piglets will increase the oxidation process in the body, resulting in the release of a large number of free radicals in the body. Excessive free radicals will cause oxidative reaction in the body, which will seriously damage the balance of the environment in the body [36,37], and damage the intestinal health. High concentrations of ROS attack the carbohydrates, lipids, proteins and nucleic acids of intestinal endothelial cells and epithelial cells, leading to oxidative damage of intestinal cells and affecting the structure and function of the intestinal tract. MDA is a metabolite of lipid oxidation [38], and is widely used as an important marker of oxidative stress. GSH-Px is a key enzyme antioxidant, which protects cells from oxidative damage by reducing the rising state of oxidative stress, eliminating the toxicity of peroxide and converting it into non-toxic substances [39]. This study showed that the contents of ROS, MDA and GSH-Px in the music group had no significant changes compared with the control group. The contents of ROS and MDA in the noise group were significantly higher than those in the control group and music group, and the content of GSH-Px was significantly decreased. Under the stimulation of harmful factors, the excessive production of ROS or the weakening of antioxidant capacity of the body will lead to oxidative stress [21,40]. Our results show that noise stimulation leads to oxidative stress; this is consistent with previous research in which both short term and long-term noise stimulation caused oxidative stress in the body [41,42]. The results of this study indicate that Mozart music has no significant effect on the changes in jejunal oxidase in piglets caused by transportation, but noise can exacerbate oxidative stress response of transport on the jejunum of piglets. This study speculated that noise increased the release of cortisol by activating the HPA axis, thereby exacerbating the oxidative stress response of transport to the jejunum of piglets.

### Effects of music and noise treatment on jejunal barrier of piglets after transportation

The gut is an important tissue for digestion, absorption and nutrient metabolism [43], and can easily become a target organ for adverse reactions such as heat, transport and hypoxia [44]. When the body is in a state of transport stress, it can easily damage the mucosal barrier of intestinal epithelial cells, which may cause the rupture and shedding of intestinal villous epithelial cells, or further induce changes in intestinal tissue morphology [45]. Villus height, crypt depth and ratio of villus to crypt are important indicators to evaluate intestinal morphology, which can reflect intestinal function and health status to a certain extent. The increase of intestinal villi height is beneficial to improve the absorption function of small intestine, which reflects the integrity of intestinal structure [46]. The villus to crypt ratio reflects the rate of renewal of the intestinal epithelial cells, with higher ratios indicating faster renewal and a healthier intestinal tract. At the same time, intestinal permeability is also an extremely important evaluation index of intestinal barrier function. Diamine oxidase (DAO) is a highly active intracellular enzyme present in the upper villi of the small intestine mucosa in mammals. DAO plays an important role in the metabolism of histamine and many polyamines, and the activity of this enzyme is closely related to the synthesis of nucleic acid and protein in mucosal cells. DAO can reflect the integrity and degree of damage to the intestinal mechanical barrier. Similar to DAO, when the intestinal barrier is damaged, the D-lactic acid (DLA) content also increases, reflecting the integrity of the intestinal tract. The activity levels of DLA and DAO in blood can reflect intestinal permeability [47]. When the intestinal mucosa is damaged, the intestinal permeability increases, and the plasma levels of DLA and DAO activities will increase [47]. In this study, compared with the CON group piglets, there were no significant changes in the villus length and crypt depth of the jejunum in the MUS group piglets. Compared with the CON and MUS groups, the NOI group had reduced jejunal villus length and crypt depth. In addition, the plasma levels of DLA and DAO in the MUS group were significantly lower than those in the CON and NOI groups, while the plasma levels of DLA and DAO in the NOI group were significantly higher. This indicates that music processing has a protective effect on the integrity of the intestinal tract in piglets, reducing the damage caused by transportation to the intestinal mucosa, while noise damages the intestinal mucosa and reduces intestinal permeability. Previous studies have shown that the increase in cortisol caused by stress can cause intestinal inflammation and oxidative stress through the brain-gut axis. Stress can lead to rapid degradation of intestinal structure, induce atrophy of intestinal villi, and increase the chance of secondary infection through damaged intestinal barrier function [2,48]. It is speculated that the protective effect of Mozart’s music on intestinal tract may be to reduce the activity of HPA axis, lead to the decrease of serum cortisol concentration and inflammatory reaction, and thus reduce the damage to intestinal tract caused by transportation. Noise stimulation can activate the activity of HPA axis, which leads to the increase of serum cortisol concentration and oxidative stress, thus aggravating the intestinal oxidative damage caused by transportation stress. This is consistent with previous studies, in which music reduced the concentration of cortisol, while noise increased the cortisol concentration, reduced intestinal permeability, and caused intestinal oxidative stress [49].

### Correlation analysis of music and noise treatment on the jejunum inflammation and oxidative stress and transcriptome of piglets after transportation

In order to study the changes of gene level, gene function and gene products in the jejunum of piglets after transportation under music and noise treatment, the DEGs in the jejunum were determined by transcriptome sequencing method, and the DEGs in the jejunum were analyzed by KEGG database. According to the detection of physiological indexes of the jejunum in piglets, this study found that music treatment could reduce the level of cortisol in piglets and reduce the inflammatory damage of the jejunum caused by transport stress; noise increased the cortisol level of piglets, and aggravated the oxidative stress level of piglets following transportation. The transcriptome results showed that the KEGG pathways “NF kappa B signaling pathway”, “PI3K Akt signaling pathway” and “B cell receptor signaling pathway” related to inflammation were enriched in the jejunum of piglets in MUS group and CON group. However, the “PI3K Akt signaling pathway”, “cancer pathway” and other KEGG pathways in the jejunum of piglets in NOI group and CON group were enriched. The activation of “NF kappa B signaling pathway” [50], “PI3K Akt signaling pathway” [51,52], “Bcell receptor signaling pathway” [53] and “cancer pathway” are closely related to inflammation and oxidative stress, in which NF kappa B is a key transcription factor that initiates inflammation and regulates a variety of cytokines, including TNF-a, IL-1b, IL-6 and IL-18. The genes PIK3 CD, LYN, VAV1, CD22, IRAK2 and FOXO1 used to verify the accuracy of transcription, as expected, were significantly down-regulated in the jejunum of MUS group. According to the genecards official website, these genes are related to inflammation or oxidative stress, which indicates that the mRNA expression level of inflammatory genes in the jejunum of piglets in MUS group was decreased, and the expression of genes related to oxidative stress in the jejunum of piglets in NOI group was increased. Therefore, it is speculated that Mozart music may reduce serum cortisol concentration and jejunal inflammatory gene expression by regulating the activity of HPA axis, thereby inhibiting jejunal inflammatory pathway and inflammatory response of piglets in MUS group, and alleviating jejunal stress injury induced by transportation. However, noise caused oxidative damage to the jejunum of piglets by activating the HPA axis, releasing cortisol, activating the genes and pathways related to oxidative stress in the jejunum, and causing oxidative damage to the jejunum of piglets by noise.

## Conclusion

Mozart music stimulation reduced the increase of cortisol concentration caused by transportation in piglets by reducing the activity of HPA axis, and alleviated the inflammatory reaction of of piglets’ intestinal tract during transportation. Noise increased the activity of HPA axis, aggravated the damage of stress on the intestinal tract of piglets, and caused oxidative damage to the intestinal tract of piglets. This study explored the role and mechanism of music in alleviating intestinal injury caused by transportation stress in piglets. It is confirmed that Mozart’s music therapy can have a positive impact on the welfare of piglets and provide an important theoretical basis for improving the welfare of piglets during transportation. Our results provide a new idea for alleviating the stress response caused in piglets during transportation. We provided a way to study the transportation welfare of piglets.

## Abbreviations

HPA: the hypothalamus pituitary adrenal cortex
CRH: corticotropin releasing hormone
PVN: hypothalamic paraventricular nucleus
ACTH: adrenocorticotropic hormone
DLA: D-lactic acid
DAO: diamine oxidase
IL-6: interleukin 6
IL-8: interleukin 8
IL-12: interleukin 12
ROS: reactive oxygen species
MDA: malondialdehyde
GSH-PX: glutathione peroxidase
DEGs: differentially expressed genes
KEGG: Kyoto Encyclopedia of Genes and Genomes
